# Maternal Heart Rate Variability during the First Stage of Labor

**DOI:** 10.3389/fphys.2017.00774

**Published:** 2017-10-09

**Authors:** Shaza M. Musa, Ishag Adam, Nada G. Hassan, Duria A. Rayis, Mohamed F. Lutfi

**Affiliations:** ^1^Faculty of Medicine, Alneelain University, Khartoum, Sudan; ^2^Faculty of Medicine, University of Khartoum, Khartoum, Sudan; ^3^College of Medicine, Qassim University, Buraydah, Saudi Arabia

**Keywords:** cardiac autonomic modulations, heart rate variability, labor, sympathovagal, Sudan

## Abstract

Labor necessitates continuous adjustments of cardiac autonomic reflexes by alternate activation of the sympathetic and parasympathetic nervous systems. The division of the autonomic nervous system (ANS) that predominates during the first stage of labor is unclear and needs to be further investigated. The study aimed to compare heart rate variability (HRV) in pregnant women in the third trimester with those during the first stage of labor. We conducted a case–control study at Saad Abul Ela Maternity Hospital, Khartoum, Sudan. Forty-five women with singleton, live neonates in the first stage of labor and 45 women in the third trimester (but not in labor) were enrolled as case and control groups, respectively. Data on the medical history, obstetrics history, and clinical examinations that were performed in all of the studied women were obtained using prearranged questionnaires. Cardiac autonomic modulation (CAM) of the heart was examined in both groups based on time and frequency domain HRV indices. There were no significant differences in age, parity, body mass index, and hemoglobin levels between the two groups. Pregnant women in labor had significantly higher LnSDNN, LnRMSSD, LnTP, LnVLF, LnLF, LnHF, LF Norm, and LnLF/HF ratio, but lower HF Norm compared with controls (*P* < 0.001). These findings remained unchanged when possible confounders were controlled for using regression analysis. Our findings suggest a significant increase in indictors of sympathetic CAM, namely LF Norm and LnLF/HF, during labor. Sympathetic hypertonia associated with labor is unlikely to increase the risk of cardiac events because sympathetic CAM simultaneously increases with global HRV. Increased HRV during labor may be explained by parasympathetic activation as indicated by higher LnHF and LnRMSSD at the time of delivery.

## Introduction

Labor is associated with significant physiological changes, most of which are mediated by the autonomic nervous system (ANS) (Sanghavi and Rutherford, 2014; Soma-Pillay et al., 2016). During the first stage of labor, there is considerable fluctuation in cardiac preload because of cyclic variations in venous return to the heart (Robson et al., 1987). Up to half a liter of blood shifts from the uterine/placental circulation to the systemic circulation during uterine contractions (UCs) and vice versa during uterine relaxation (UR) (Sanghavi and Rutherford, 2014). This necessitates continuous adjustments of cardiac autonomic reflexes by alternate activation of the sympathetic and parasympathetic nervous systems. To keep cardiac preload equal to afterload, sympathetic discharge to the heart increases when venous return increases during UCs. Alternatively, parasympathetic discharge to the heart increases when venous return decreases during UR. Theoretically, both divisions of the ANS may be activated during each UC by mechanisms other than fluctuation of cardiac preload (Jones and Greiss, 1982; Ronca et al., 2006; Norman et al., 2011; Gamer and Büchel, 2012; Reyes-Lagos et al., 2015). Pain and anxiety associated with UCs induce secretion of catecholamines and consequently induce generalized sympathetic discharge (Jones and Greiss, 1982; Ronca et al., 2006). However, the hormone oxytocin, which is released during labor to induce UCs, also augments parasympathetic cardiac autonomic modulation (CAM) (Norman et al., 2011; Gamer and Büchel, 2012; Reyes-Lagos et al., 2015). Therefore, which division of the ANS predominates during the first stage of labor is unclear.

Previous studies that have examined sympathovagal balance during the first stage of labor are limited and were primarily designed to evaluate CAM during UCs (Reyes et al., 2011; Suzuki et al., 2012; Reyes-Lagos et al., 2015; Gonçalves et al., 2016). These studies were largely based on low frequency (LF) and high frequency (HF) power spectral density and lacked detailed assessment of the overall heart rate variability (HRV) parameters (Reyes et al., 2011; Suzuki et al., 2012; Reyes-Lagos et al., 2015; Gonçalves et al., 2016).

Therefore, the present study aimed to compare CAM in pregnant women in the third trimester with those during the first stage of labor. Detailed time and frequency domains of HRV were considered to assess the risk of cardiac events during the first stage of labor.

## Materials and methods

The present case–control study was conducted at Saad Abul Ela Maternity Hospital, Khartoum, Sudan during July to December 2016. This study was carried out in accordance with the recommendations of the research committee (Research Board of Faculty of Medicine, Alneelain University), Sudan, and written informed consent was obtained from all of the women. The study was performed in accordance with the Declaration of Helsinki.

The cases were women with singleton, live neonates in the first stage of labor. The controls were women in the third trimester, but they were not in labor. Women with hypertension, diabetes mellitus, heart disease or any other medical condition complicating pregnancy were excluded from the study.

After signing informed consent, information on the medical and obstetrics history (age, parity, gestational age) was gathered from cases and controls using prearranged questionnaires. Labor was diagnosed by the presence of regular uterine contractions using cardiotocography and cervical dilatation on a pelvic examination (Hanley et al., 2016). Weight and height were measured and body mass index (BMI) was calculated via the following equation: BMI (kg/m^2^) = weight in kilograms/height in meters^2^. Following complete bed rest for 10 min, systolic (SBP) and diastolic (DBP) blood pressure was measured and HRV recording was obtained using the Biocom 3000 recorder (Heart Rhythm Scanner, version 2.0; Biocom Technologies) (Task Force of the European Society of Cardiology and the North American Society of Pacing and Electrophysiology, 1996). Women were advised to lie down (in the supine position) and breathe comfortably while collecting data. Electrocardiography (ECG) recording was initiated ensuring clean ECG signals and an absence of movement artifacts. The Heart Rhythm Scanner automatically finished the trial session once its time expired (5 min). After recording the session, the ECG data were reviewed for abnormal ECG readings. The Heart Rhythm Scanner software is capable of performing an automatic search for various types of abnormal ECG recordings irrelevant to their cause. This is based on the standard statistical procedure of exclusion of rough artifacts from the data series. Abnormal ECG readings were deleted and the software was allowed to calculate HRV parameters from the rest of the raw data. Autonomic modulation of the heart during labor was examined using time and frequency domain analysis. The natural logarithm (Ln) was used to express time domain HRV parameters, such as the standard deviation of the NN interval (LnSDNN), the square root of the mean squared differences of successive NN intervals (LnRMSSD), and frequency domain HRV parameters, such as total power (LnTP), very low frequency (LnVLF), low frequency (LnLF), and high frequency (LnHF) power spectral density. The frequency ranges of VLF, LF, and HF were 0.0033–0.04, 0.039–0.15, and 0.149–0.4 Hz, respectively (Task Force of the European Society of Cardiology and the North American Society of Pacing and Electrophysiology, 1996). Sympathetic and parasympathetic CAMs were evaluated by normalized low frequency (LF Norm) and high frequency (HF Norm), respectively (Lutfi, 2011, 2012, 2015; Musa et al., 2016). The LnLF/HF ratio was used to assess sympathovagal autonomic balance of the heart. LnSDNN and LnTP were used to assess overall HRV and RMSSD as an index of vagal modulation. LF Norm and HF Norm were calculated using the following formulae: LF Norm (nu) = $\frac{\text{LF }\times \;100}{\text{TP }-\text{ VLF}}$ and HF Norm (nu) = $\frac{\text{HF}\times 100}{\text{TP }-\text{ VLF}}$, respectively (Lutfi, 2011, 2012, 2015; Musa et al., 2016).

HRV, similar to many other physiological parameters, is subjected to a circadian rhythm (Jensen et al., 2016). HRV was measured in this study in the morning between 8:00 a.m. and 12 p.m. to avoid the confounding effect of circadian rhythm on CAM.

A sample size of 45 women in each arm of the study groups (cases and controls) was calculated to provide a significant difference in the mean of the HRV parameters with 80% power and a difference of 5% at α = 0.05.

Data were entered in a computer and analyzed using SPSS for Windows version 20.0 (SPSS Inc., Chicago, IL, USA). Continuous and categorical data were compared between the cases and controls using the *t*-test and chi-square test, respectively. Linear regression analyses were performed where Ln of HRV parameters were the dependent variables and sociodemographic data (age, parity, gestational age), BMI, hemoglobin, and labor were the independent variables. Heart rate is inversely proportional to NN intervals, from which all HRV parameters are derived. Regression analysis was used to adjust for variation in the mean heart rate when associations between labor and HRV indices were assessed. *P* < 0.05 was considered significant.

## Results

There were no significant differences in age, gestational age, BMI, hemoglobin concentrations, SBP, DBP, and mean heart rate between pregnant women in labor and controls (Table 1).

**Table 1:** Characteristic of the study groups.

	**Pregnant women during labor**	**Control group**	***P***
	**(*n* = 45)**	**(*n* = 45)**	
Age, years	28.2 (5.1)	28.9 (4.9)	0.535
Parity	1.6 (1.8)	1.8 (2.0)	0.746
Gestational age, weeks	38.6 (2.0)	38.5 (0.6)	0.730
BMI, kg/m^2^	29.0 (3.3)	30.9 (6.0)	0.069
Hemoglobin, g/dl	11.6 (0.9)	11.8 (1.0)	0.448
SBP, mm/Hg	115.5 (9.9)	115.8 (9.1)	0758
DBP, mm/Hg	75.4 (6.2)	74.6 (7.0)	0.581
MHR (Beats/min)	93.8 (9.9)	96.6 (11.7)	0.752

**Figure 1:**
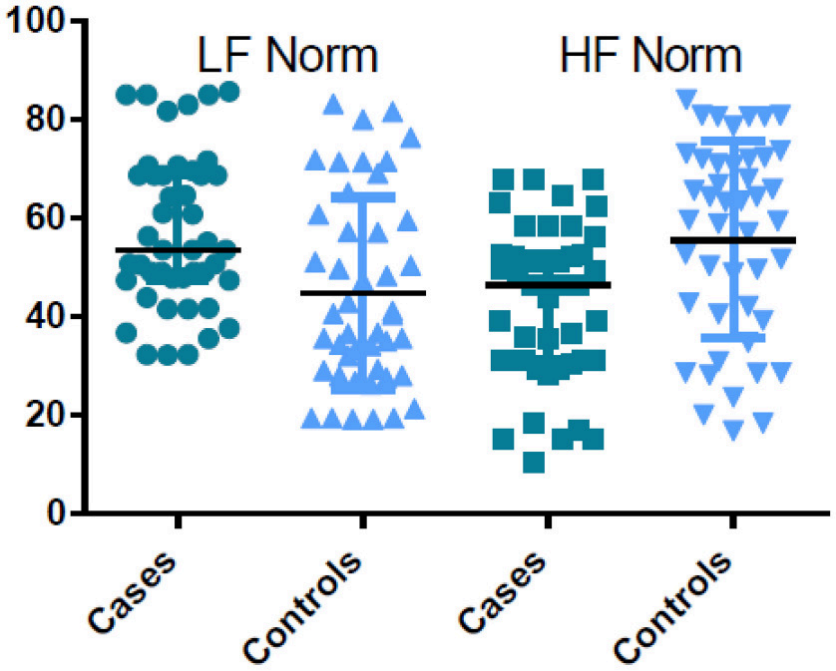
Comparison of LF norm and HF norm between the cases and controls.

Pregnant women in labor had significantly higher time and frequency domain HRV indices compared with controls, except for HF Norm. HF Norm was lower in pregnant women in labor than in controls (Table 2, Figure 1).

**Table 2:** Comparison of time and frequency domains HRV indices between women during labor and the control group.

**Variables**	**Pregnant women during labor**	**Control group**	***P***	***P***
	***N* = 45**	***N* = 45**	**Not adjusted for MHR**	**Adjusted for MHR**
LnSDNN	4.43 (0.98)	3.51 (0.54)	<0.001	<0.001
LnRMSSD	4.47 (1.13)	3.61 (0.61)	<0.001	<0.001
LnTP (ms^2^/Hz)	7.44 (2.46)	5.04 (1.04)	<0.001	<0.001
LnVLF (ms^2^/Hz)	6.40 (2.53)	4.03 (1.02)	<0.001	<0.001
LnLF (ms^2^/Hz)	6.32 (2.44)	3.57 (1.06)	<0.001	<0.001
LnHF (ms^2^/Hz)	5.64 (1.89)	3.79 (1.31)	<0.001	<0.001
LF Norm (nu)	57.05 (15.64)	44.75 (19.43)	0.001	<0.001
HF Norm (nu)	42.67 (15.08)	55.65 (15.84)	0.001	<0.001
LnLF/HF	0.67 (1.65)	−0.22 (0.86)	0.002	<0.001

*HF, high frequency; HF, Norm normalized high frequency; HRV, heart rate variability; LF, low frequency; LF, Norm normalized low frequency; Ln, natural logarithm; MHR, mean heart rate; RMSSD, square root of the mean squared differences of successive NN intervals; SDNN, standard deviation of the NN intervals; TP, total power; VLF, very low frequency*.

**Figure 2:**
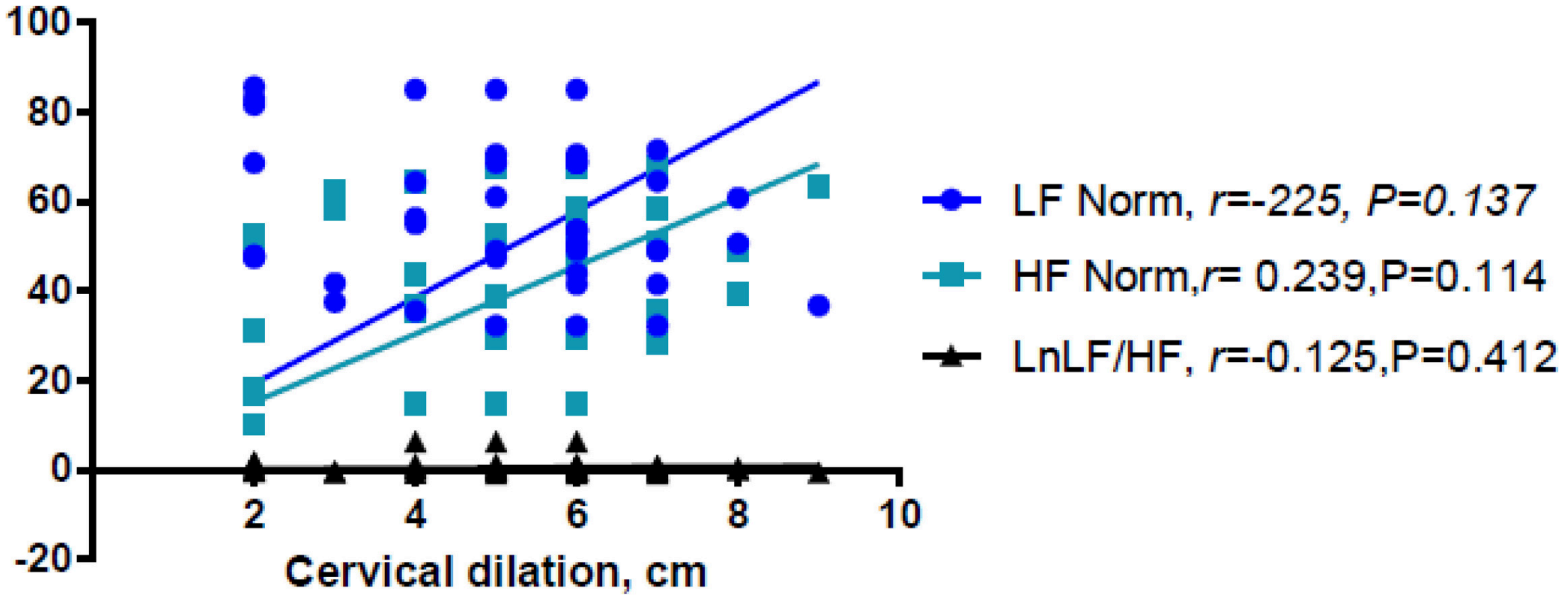
Correlations between cervical dilation and LF Norm, HF Norm, and LnLF/HF.

Linear regression analysis showed significant positive associations between labor and LnVLF, LnLF, and LnHF (Table 3). There were no significant correlation between cervical dilation (in women in labor) and LF Norm (nu), HF Norm (nu), and LnLF/HF (Figure 2). Additionally, labor was positively associated with LF Norm and LnLF/HF, but negatively associated with HF Norm (Table 4).

**Table 3:** Factors associated with LnVLF, LnLF (ms^2^/Hz), and LnHF in the groups using linear regression analyses.

	**Ln VLF (ms**^**2**^**/Hz)**			**LnLF (ms**^**2**^**/Hz)**			**LnHF (ms**^**2**^**/Hz)**		
	***CE***	***SE***	***P***	***CE***	***SE***	***P***	***CE***	***SE***	***P***
Age, year	−0.040	0.039	0.307	−0.074	0.041	0.076	−0.038	0.036	0.303
Gravidity	−0.140	0.116	0.228	−0.154	0.112	0.172	−0.148	0.097	0.134
Parity	−0.041	0.101	0.688	−0.084	0.106	0.428	−0.090	0.093	0.337
Gestational age, weeks	0.067	0.125	0.591	−0.044	0.131	0.739	−0.189	0.115	0.104
BMI, (kg)/(m)^2^	−0.016	0.038	0.669	0.008	0.040	0.849	0.002	0.035	0.950
Hemoglobin, g/dl	0.136	0.186	0.468	0.192	0.195	0.327	0.032	0.172	0.853
Labor^*^^†^	2.254	0.358	<0.001	2.690	0.374	<0.001	<1.801	0.329	0.000
MHR (Beats/min)	−0.094	0.017	<0.001	−0.065	0.018	<0.001	−0.054	0.016	0.001

*HF, high frequency; LF, low frequency; Ln, natural logarithm; MHR, mean heart rate; SE, standard error; VLF, very low frequency*.

* *Adjusted for MHR*.

† *The cases according to cervical dilation*.

**Table 4:** Factors associated with LF Norm, HF Norm, and LnLF/HF in the groups using linear regression analyses.

	**LF Norm (nu)**			**HF Norm (nu)**			**LnLF/HF**		
	***CE***	***SE***	***P***	***CE***	***SE***	***P***	***CE***	***SE***	***P***
Age, year	−0.510	0.412	0.219	0.429	0.420	0.310	−0.036	0.032	0.253
Gravidity	1.240	1.050	0.241	−1.044	1.074	0.334	−0.006	0.080	0.937
Parity	0.957	1.059	0.369	−0.721	1.081	0.506	0.006	0.081	0.945
Gestational age, weeks	2.523	1.309	0.057	−2.593	1.335	0.056	0.145	0.100	0.151
BMI, (kg)/(m)^2^	0.373	0.403	0.356	−0.401	0.411	0.332	0.005	0.031	0.861
Hemoglobin, g/dl	2.112	1.950	0.282	−1.774	1.989	0.375	0.161	0.150	0.286
Labour^*^^†^	13.063	3.745	0.001	−13.77	3.821	0.001	0.889	0.287	0.003
MHR (Beats/min)	0.266	0.179	0.141	−0.303	0.183	0.101	−0.011	0.014	0.413

*HF, Norm normalized high frequency; LF, Norm normalized low frequency; SE, standard error*.

* *Adjusted for MHR*.

† *The cases according to cervical dilation*.

## Discussion

The current study showed that during labor (i) sympathetic and parasympathetic tone increased (as shown by LF Norm and RMSSD values), and (ii) there was a shift in sympathovagal balance toward a larger sympathetic predominance (as shown by LF/HF values). To the best of our knowledge, the present study is the first to examine CAM during labor based on detailed time and frequency domain HRV analysis. However, a few previous reports are relevant to the present study (Reyes et al., 2011; Suzuki et al., 2012; Gonçalves et al., 2016). In a recent study, Gonçalves et al. measured maternal HRV during labor using the two techniques of ECG and photoplethysmography (PPG) (Gonçalves et al., 2016). The primary objective of Gonçalves et al.'s study was to evaluate the effect of signal loss on maternal HRV indices when PPG was used instead of ECG for measuring heart rate. They showed that LF Norm was > HF Norm and LF/HF was > 1 when ECG was used to assess HRV at 1 h (95% confidence interval of LF Norm = 45.28–50.38 nu, HF Norm = 28.33–32.18 nu, LF/HF = 1.47–1.69) and 2 h (95% confidence interval of LF Norm = 43.29–49.99 nu, HF Norm = 28.03–32.48 nu, LF/HF = 1.35–1.67) before childbirth. When PPG was used to record heart rate, the readings of LF Norm, LF Norm, and LF/HF were twice, one-sixth and 10-fold, respectively, compared with those of ECG. Accordingly, PPG and ECG findings suggested predominance of sympathetic CAM during labor. Owing to changing dynamics during the intrapartum period, HRV evaluated by Gonçalves et al. over 1 h was higher compared with the present study, which was based on 5-min ECG recordings. This previous study is further supported by findings of Suzuki et al when they assessed maternal HRV in 20 pregnant women during non-complicated labor and another 15 pregnant women who were threatened with premature labor contractions (Suzuki et al., 2012). In both groups, absolute values of VLF and LF power were higher, while HF power was comparable, during UCs compared with the periods between UCs. Additionally, LF/HF significantly increased during UCs, which suggested upregulated maternal sympathetic CAM during these periods (Suzuki et al., 2012).

Absolute HF power is commonly used as an indicator of parasympathetic CAM. However, reliability of absolute LF power as a sympathetic CAM index is debatable (Reyes del Paso et al., 2013). Absolute LF power is affected by the baroreflex and thus may also reflect parasympathetic CAM (Goldstein et al., 2011). Some authors preferred LF Norm to evaluate sympathetic CAM (Lutfi, 2015). The use of LF Norm minimizes the effects due to variations in VLF and thus better reflects sympathetic CAM (Lutfi, 2011).

In the present study, LnLF and LnHF were proportionally higher in pregnant women during labor compared with controls. In contrast, comparison between the groups showed a reciprocal change in LF Norm and HF Norm (i.e., labor resulted in increased LF Norm, but a simultaneous decrease in HF Norm). The reciprocal pattern of change in LF Norm and HF Norm, but not their absolute values, suggests that normalized indices are better indicators for sympathetic and parasympathetic CAM (Lutfi, 2011, 2012, 2015). This might be because when discharge of one division of the ANS (sympathetic or parasympathetic) increases, the tone of the other division is likely to decrease.

When parasympathetic CAM predominates, global HRV increases, leading to a lower risk of cardiac events (Task Force of the European Society of Cardiology and the North American Society of Pacing and Electrophysiology, 1996; Lutfi, 2011). However, there are exceptions to this situation. One exception is that poor HRV was repeatedly demonstrated in patients with bronchial asthma, despite augmented parasympathetic CAM among the affected patients (Gupta et al., 2012; Lutfi, 2012, 2015). Another exception was shown in the present study, where sympathetic CAM (LF Norm, LnLF/HF) was obviously enhanced during labor together with global HRV (LnSDNN and LnTP) compared with controls. The explanation for these exceptions is unknown and requires further research and investigations.

Two implications are essential regarding increased time and frequency domain HRV indices during labor. First, predominance of sympathetic CAM during labor, as suggested by the findings of LF Norm, HF Norm, and LF/HF, does not definitely indicate complete withdrawal of parasympathetic CAM. In our study, LnRMSSD and LnHF were significantly augmented during labor, which represent evidence of parasympathetic activation during this physiological process. Several previous reports have shown the importance of parasympathetic activation during labor. In proinflammatory conditions, such as labor (Houben et al., 2009), acetylcholine is released as a result of parasympathetic stimulation to suppress certain inflammatory cytokines, and consequently protects against exaggerated tissue damage (Huston and Tracey, 2011; Andersson and Tracey, 2012; Reyes-Lagos et al., 2015). Oxytocin released during labor also augments parasympathetic CAM (Norman et al., 2011; Gamer and Büchel, 2012; Reyes-Lagos et al., 2015). However, stress associated with pain and anxiety during labor induces secretion of catecholamines and thus generalized sympathetic discharge (Jones and Greiss, 1982; Ronca et al., 2006). Squeezing of blood into the general circulation during UCs increases venous return to the heart and activates several cardiovascular reflexes mediated by the sympathetic nervous system (Robson et al., 1987; Sanghavi and Rutherford, 2014; Soma-Pillay et al., 2016). Therefore, both divisions of the ANS appear to be activated during labor. Sympathetic CAM is more obvious because of substantial changes in hemodynamics and other stresses associated with labor. This explains the significant increase in indictors of sympathetic CAM, such as LF Norm and LnLF/HF, during labor. However, parasympathetic activation due to release of oxytocin and the anti-inflammatory cholinergic response elicited by labor are also shown by findings of increased LnHF and LnRMSSD (Reyes-Lagos et al., 2015).

Second, enhanced sympathetic CAM during labor is unlikely to result in hazardous cardiac events. In conditions such as myocardial infarction, increased sympathetic discharge to the heart depresses HRV, placing affected patients at higher risk of adverse scenarios, such as arrhythmia and cardiac arrest. Theoretically, sympathetic hypertonia associated with labor is unlikely to behave like myocardial infarction because sympathetic CAM increases simultaneously with global HRV. However, this possibility remains hypothetical until proven by further investigations and studies.

## Conclusions

The findings of the present study suggest that both divisions of the ANS are activated during labor. The substantial changes in hemodynamics, pain and anxiety associated with UC are likely explanations for the significant increase of the indictors of sympathetic CAM, namely, LF Norm and LnLF/HF, during labor. Alternatively, parasympathetic activation due to release of oxytocin and the anti-inflammatory cholinergic response may explain why LnHF and LnRMSSD were increased during labor. Sympathetic hypertonia associated with labor is unlikely to increase the risk of cardiac events because sympathetic CAM increases simultaneously with global HRV. However, this implication remains hypothetical until proved by further investigations and research.

## Author contributions

IA designed the study. SM, NH, and DR carried out experimental protocols. IA and ML analyzed the data. ML and IA prepared and revised the manuscript. All authors read and approved the final manuscript.

### Conflict of interest statement

The authors declare that the research was conducted in the absence of any commercial or financial relationships that could be construed as a potential conflict of interest.
